# A comparative analysis of the properties of regulated promoter systems commonly used for recombinant gene expression in *Escherichia coli*

**DOI:** 10.1186/1475-2859-12-26

**Published:** 2013-03-18

**Authors:** Simone Balzer, Veronika Kucharova, Judith Megerle, Rahmi Lale, Trygve Brautaset, Svein Valla

**Affiliations:** 1 Department of Biotechnology, NTNU, Sem Sælands vei 6, Trondheim, 7491, Norway; 2 Faculty of Physics and CeNS, LMU Munich, Munich, 80539, Germany; 3 Department of Biotechnology, SINTEF Materials and Chemistry, Trondheim, 7465, Norway

**Keywords:** Recombinant expression, Regulator/promoter systems, XylS/*Pm*, XylS/*Pm* ML1-17, LacI/*P*_*T7lac*_, LacI/*P*_*trc*_, AraC/*P*_*BAD*_, Systematic comparison

## Abstract

**Background:**

Production of recombinant proteins in bacteria for academic and commercial purposes is a well established field; however the outcomes of process developments for specific proteins are still often unpredictable. One reason is the limited understanding of the performance of expression cassettes relative to each other due to different genetic contexts. Here we report the results of a systematic study aiming at exclusively comparing commonly used regulator/promoter systems by standardizing the designs of the replicon backbones.

**Results:**

The vectors used in this study are based on either the RK2- or the pMB1- origin of replication and contain the regulator/promoter regions of XylS/*Pm* (wild-type)*,* XylS/*Pm* ML1-17 (a *Pm* variant), LacI/*P*_*T7lac*_*,* LacI/*P*_*trc*_ and AraC/*P*_*BAD*_ to control expression of different proteins with various origins. Generally and not unexpected high expression levels correlate with high replicon copy number and the LacI/*P*_*T7lac*_ system generates more transcript than all the four other cassettes. However, this transcriptional feature does not always lead to a correspondingly more efficient protein production, particularly if protein functionality is considered. In most cases the XylS/*Pm* ML1-17 and LacI/*P*_*T7lac*_ systems gave rise to the highest amounts of functional protein production, and the XylS/*Pm* ML1-17 is the most flexible in the sense that it does not require any specific features of the host. The AraC/*P*_*BAD*_ system is very good with respect to tightness, and a commonly used bioinformatics prediction tool (RBS calculator) suggested that it has the most translation-efficient UTR. Expression was also studied by flow cytometry in individual cells, and the results indicate that cell to cell heterogeneity is very relevant for understanding protein production at the population level.

**Conclusions:**

The choice of expression system needs to be evaluated for each specific case, but we believe that the standardized vectors developed for this study can be used to more easily identify the nature of case-specific bottlenecks. By then taking into account the relevant characteristics of each expression cassette it will be easier to make the best choice with respect to the goal of achieving high levels of protein expression in functional or non-functional form.

## Background

Parameters affecting recombinant protein expression in *Escherichia coli* have been studied extensively and numerous methods aiming at improving protein yields have been reported, usually involving genetic manipulations and/or production process optimization [1-4]. However, in spite of the large number of potentially useful approaches available there is still no guarantee that a satisfactory result will be obtained in each specific case, and trial and error is therefore currently an integrated part of development of new protein production processes. The work involved in this can become very laborious since many parameters such as choice of strains, vector construct designs, growth media and cultivation conditions can potentially have a big and unpredictable effect on the process. Steadily more promoter systems for regulated protein expression in *E. coli* ([1] and references therein, [2-6]) are being developed, increasing the complexity. The studies of those novel expression systems were commonly based on experiments involving vectors with different backbones [2,4,7,8]; typically commercially available and commonly used vectors from the pET [9], pTrc [10] or pBAD [11] series. More theoretical approaches have also been used [6,12]. However, expression is influenced by many parameters even within vectors, like the presence or absence of sequences of the 5^′^ coding region encoding N-terminal fusion partners (His_6_ tag [13], N-terminal signal peptides [14], and others), different origins of replication [15-17], different terminators [18] or selection markers. Penicillins for example are very frequently used for selection in spite of their known rapid degradation due to secreted β-lactamase [19]. A first step towards a more systematic, backbone-independent approach is described in a study performed by Tegel et al. [20] in which expression from three different IPTG-inducible promoters (*P*_*T7lac*_, *P*_*trc*_, *P*_*lac*_) is compared. These are all based on the negative regulator LacI, while positively regulated promoters such as *P*_*BAD*_ and *Pm* have not been used in such comparative studies. The regulators of these two promoters (AraC and XylS, respectively) are both members of the same family of transcriptional activators [21]. The AraC*/P*_*BAD*_ system is quite extensively used and its characteristics have been reviewed [1]. The XylS*/Pm* system was included because it has several beneficial traits for protein expression in general (reviewed by Brautaset et al. [21]), and in combination with RK2 minimal replicons it has been demonstrated to be capable of expressing proteins at industrial levels in high cell density cultivations [14,22], We have used this system extensively in our laboratory as a model for studies of recombinant gene expression. Particular advantages of this system are that the levels of expression can be fine-tuned by various means [23-25], that it is not host-dependent in contrast to most other systems and that the inducer is cheap. Furthermore, expression from the native system could be greatly improved by generating variants of the regulator protein XylS [26], the DNA region corresponding to the *Pm* promoter region [27] as well as the region corresponding to the *Pm* 5^′^-untranslated region (5^′^-UTR) [28].

In this report we describe a systematic comparison of both positively and negatively regulated expression systems. Being aware of the influence of the 5^′^ end of the coding region on expression [29,30], we intentionally chose to use model genes with native 5^′^ ends as opposed to commonly used regions encoding N-terminal detection tags or solubility-enhancing fusion partners. The expression analyses were carried out at both the transcript and the protein level (activity assays and total protein), and we also included a flow cytometry based analysis of expression in individual cells. All comparisons were performed using identical vector backbones, a procedure we believe can be used generally as a diagnostic tool to identify bottlenecks in recombinant protein production processes.

## Results and discussion

### Construction of a set of plasmids specifically designed for comparative studies of commonly used expression systems in *E. coli*

**Figure 1:**
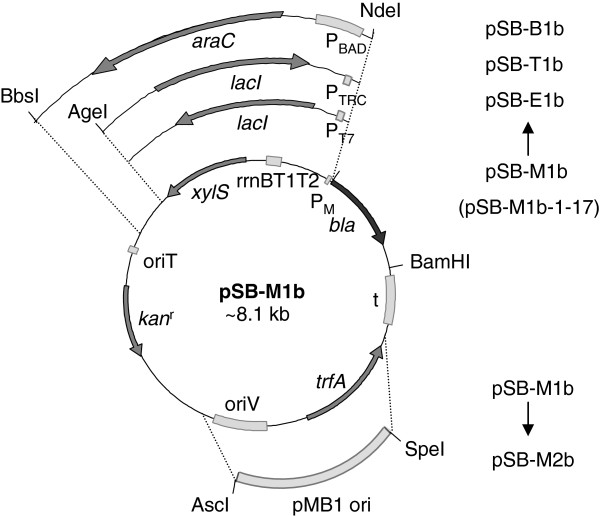
**Illustration showing how the different constructs in the study were generated based on pSB-M1b.** The upper part shows how the alternative regulator/promoter systems were incorporated. pSB-M1b-1-17 contains a variant of the *Pm* core promoter termed ML1-17 (see text). The lower part shows the *oriV/trfA* region in pSB-M1b that was replaced with the pMB1 *ori* described in Table 1.

To reduce potential effects on expression unrelated to the features of the regulator/promoter systems themselves all replicons used for comparisons were designed in such a way that the backbones were identical and the expression cassettes were in all cases integrated at the same location (Figure 1 and Table 1). The selected systems include XylS/*Pm* (the native system; denoted in the figures as M); the high level expression variant *Pm* ML1-17 (abbreviated by M-1-17) [27]; LacI/*P*_*T7lac*_ originating from the pET vector series (Novagen; denoted as E); the LacI/*P*_*trc*_ system from the pTRC series of vectors (Pharmacia; denoted as T); and finally the AraC/*P*_*BAD*_ system from the pBAD series of vectors (Invitrogen, abbreviated by B). Further details related to transcriptional start sites and 5^′^-UTR regions are described in the Methods section.

**Table 1:** **Plasmids used in this study**^**a**^

**Name**	**Key features**		**Source**
pTA16/pSB-M1b^b^	*m*-toluate- inducible *Pm*, *xylS* activator gene, RK2 replicon, *bla* reporter, Kan^r^		[31]
pET16b	IPTG-inducible *P*_*T7lac*_, *lacI* repressor gene, Amp^r^		Novagen
pBAD/gIII_calmodulin	L-arabinose- inducible *P*_*BAD*_, *araC* activator gene, Amp^r^		Invitrogen
pTrc99A	IPTG- inducible *P*_*trc*_ promoter, *lacI* repressor gene, Amp^r^		Pharmacia
pIB11-*luc*_S_	pIB11 [25] with *luc*_*S*_ under control of *xylS/Pm*, Kan^r^		unpublished
pBAD24-GFP	pBAD24 with *gfpmut3* insert, Amp^r^		[32]
pHOG-173-2-5-AP	pHOG plasmid with *scFv173-2-5-phoA* fusion gene insert, provided by Affitech AS, Oslo, Amp^r^		unpublished
pMA-GH	pMA vector (GeneArt®, Invitrogen) with *GH1*_*S*_ insert, provided by Vectron Biosolutions AS, Trondheim, Amp^r^		unpublished
pMA-T-IL-1RA	pMA vector (GeneArt®, Invitrogen) with *IL1RN*_*S*_ insert, provided by Vectron Biosolutions AS, Trondheim, Amp^r^		unpublished
pSB-P0x	pSB-M1b variants with combinations of different features:		
	P… regulator/promoter system	M… *xylS/Pm*	
		M-1-17… *xylS/Pm* variant ML1-17	
		E… *lacI/P*_*T7lac*_ (from pET)	
		T… *lacI*^*q*^*/P*_*trc*_ (from pTrc)	
		B… *araC/P*_*BAD*_ (pBAD)	
	0… origin of replication	1… RK2 replicon	
		2… pMB1 replicon	
	x… reporter gene	b… *bla*	
		l… *luc*_*S*_	
		s… *scFv173-2-5-phoA*	
		g… *gfpmut3*	
		h*… GH1*_*S*_	
		r*… IL1RN*_*S*_	This study
e.g. pSB-M2l	*m*-toluate- inducible *P*_*m*_, *xylS* activator gene, pMB1 *ori*, *luc*_*S*_ reporter, Kan^r^		This study

^a^*bla:* β- lactamase gene; *luc*_S_: synthetic luciferase gene; *scFv173-2-5-phoA*: single-chain antibody fragment 173-2-5 alkaline phosphatase fusion gene; *gfpmut3*: gene for the optimized green fluorescent protein mutant 3; *GH1*_*S*_: synthetic gene for human growth hormone, *IL1RN*_*S*_: synthetic gene for human interleukin 1 receptor antagonist.

^b^ pTA16 was named pSB-M1g in this study for consistency purposes.

It is well known that gene dosage and expression levels often correlate, at least to some extent. In order to investigate any potential gene-specific effects related to this the cassettes were integrated into a mini-RK2 based replicon (pSB-M1b, 5–7 copies per cell [33]), and the pMB1 replicon (15–20 copies per cell [19], Novagen, Invitrogen) used in commercially available vectors such as pET and pBAD. In these two plasmid sets, genes coding for five different model proteins of varying biological origins were placed under control of the five promoters to cover a broad range of problems that may occur during recombinant protein production (Table 2). Note also that the use of one common N-terminal fusion tag for all proteins was avoided to study the effect of the respective promoter-5^′^-UTR regions on different 5^′^ coding sequences, as opposed to the study of Tegel et al. [20]. Specific gene sequence dependent parameters such as mRNA secondary structures and the presence of rare codons were taken into account by using optimized (for *E. coli*) synthetic genes. The corresponding genes were inserted into the two replicon types carrying the different expression cassettes, (Table 1). Not only can expression be directly compared from different regulator/promoter systems using these standardized vectors, but they can also be used more generally as tools to identify an appropriate expression system for the production of any selected target protein.

**Table 2:** Properties of the proteins selected as expression reporters

**Protein**	**Properties**
Luciferase	reporter protein, ~ 60.8 kDa, cytoplasmic localization, generally low expression, rather easy to detect, very sensitive detection via bioluminescence assay
scFv173-2-5-AP	industrially relevant protein, ~77.2 kDa, fusion protein, disulfide bonds, translocated to the periplasm, detectable through AP^a^ fusion, AP needs to be translocated to be active [34]
GFP	reporter protein, ~ 26.9 kDa, cytoplasmic localization, stable, known to be produced virtually only in its soluble form [35], very easy to detect by direct fluorometry
HGH	industrially relevant protein, ~25.1 kDa, cytoplasmic localization, usually expressed in *E. coli* as soluble protein [36,37]
IL-1RA	industrially relevant protein, ~20.1 kDa, cytoplasmic localization, usually expressed in *E. coli* as soluble protein [38,39]

^a^Alkaline phosphatase.

Due to the nature of the expression systems it was necessary to use two different *E. coli* strains as hosts. Strain ER2566 was chosen to compare expression from LacI/*P*_*T7lac*_ with XylS/*Pm* because it carries a chromosomal copy of the T7 polymerase integrated into the *lac* operon (NEB). Since the LacI/*P*_*trc*_ system is also induced by IPTG, it was decided to study expression in the same host under the assumption that the expression of T7 polymerase does not affect expression from LacI/*P*_*trc*_ due to the specificity of this polymerase for its cognate promoter [40]. Expression from XylS/*Pm* compared to AraC/*P*_*BAD*_ was performed in *E. coli* DH10B which is unable to catabolize L-arabinose, the inducer of the AraC/*P*_*BAD*_ system.

### Protein production levels are generally stimulated by increased gene dosage, but none of the tested cassettes are superior for all genes

**Figure 2:**
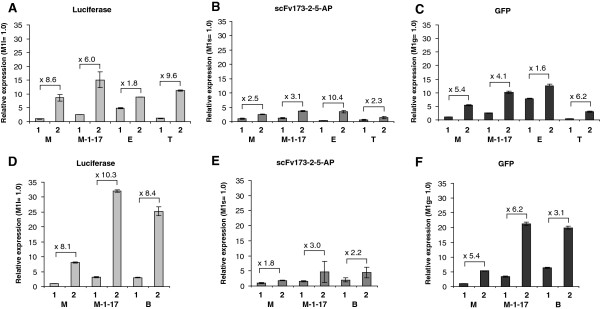
**Maximum expression of three different genes placed under control of different regulator/promoter systems.** Data represent relative expression levels under induced conditions where the activity of M1x (gene x under conrol of the *Pm* wildtype promoter, RK2 replicon) was set to 1.0. Expression was induced in a way that activity levels were maximized: 2 mM *m*-toluate for strains harboring XylS/*Pm*- based constructs, 1 mM IPTG for those with LacI/*PT7lac*, 0.2 mM IPTG for LacI/*Ptrc* and 0.015% L-arabinose for AraC/*PBAD*. The following *E. coli* strains were used as expression hosts. Panels **A**-**C**: ER2566. Panels **D**-**F**: DH10B. The naming code is the following: The capital letter represents the regulator/promoter system and the digit represents the origin of replication; for details see Table 1. The data presented are from independent biological replica.

Three different genes, encoding luciferase, an antibody fragment fused in frame to alkaline phosphatase (scFv173-2-5-AP) and green fluorescent protein (GFP), respectively, were selected as models in the initial study of the performances of the various expression cassettes (Figure 2). The alkaline phosphatase fusion protein is translocated to the periplasm, while luciferase and GFP are cytoplasmic. The results were monitored as activities, meaning that only functional proteins were measured. The only parameter that gave a consistent response for all systems was not surprisingly gene dosage, as all cassettes gave rise to more activity when they were utilized in a high plasmid copy number context. However, the fold increase was heavily protein and expression cassette dependent, ranging from 1.6 for GFP (Figure 2, Panel C) to 10.4 for the alkaline phosphatase fusion in the LacI/*P*_*T7lac*_ system (Figure 2, Panel B). We also observed that cell growth was strongly affected in several of the alkaline phosphatase fusion protein producing strains, and it was generally much more difficult to obtain reproducible data for this particular protein. We believe the reason for this is that the export of large amounts of protein is toxic to cell growth [41], in some cases also in the uninduced state. This potential toxic effect may even have resulted in accumulation of mutants that grow faster than the originally inoculated strain due to reduced scFv173-2-5-AP production. The maximal expression level is obviously very important in the context of recombinant protein production, and Figure 2 shows that in this respect none of the systems is superior for all proteins. Generally XylS/*Pm* ML1-17 and LacI/*P*_*T7lac*_ tended to produce most recombinant protein in the studies in strain ER2566 (Figure 2, Panels A-C). The mutations in the *Pm* core region were of vital importance, as XylS/*Pm* ML1-17 produced between 1.2- and 4.0- fold more active protein than the corresponding wild-type system. Note also that AraC/*P*_*BAD*_ generated similar amounts of active protein compared to XylS/*Pm* ML1-17 when the studies were done in an *ara* negative strain (DH10B; Figure 2, Panels D-F). LacI/*P*_*T7lac*_ is generally known to be a very strong system because of the efficient transcription exerted by the T7 RNA polymerase [9,40], but the comparative analysis demonstrated that this system was not superior to XylS/*Pm* ML1-17 for the genes studied here. Especially in the higher copy-number plasmids, up to four times more activity was detected in strains harboring XylS/*Pm* ML1-17. We also noted in this and other related ongoing studies in our laboratory that to get stable expression from the LacI/*P*_*T7lac*_ system, ER2566 cells needed to be freshly transformed prior to expression studies, as also recommended by Vethanayagam and Flower [42]. Similar observations were not made for any of the other three systems.

### The LacI/*P*_*T7lac*_ system is unique by its generation of large amounts of transcript and insoluble protein

**Figure 3:**
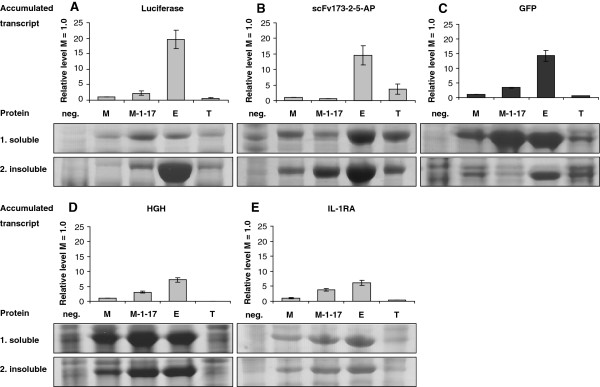
**Correlation between the accumulated transcript and protein produced after induction.** The five proteins (Panels **A**-**E**) were expressed in *E. coli* ER2566 harboring pMB1-based plasmids. Five hours after induction, samples were collected for relative quantification real-time RT-PCR (qRT-PCR) and SDS-PAGE. Accumulated transcript data were correlated to the XylS/*Pm* system (M2x; gene x under conrol of the *Pm* wildtype promoter, pMB1 replicon). The total protein fractions were separated into the soluble supernatant and the insoluble pellet fraction after sonication and separated through SDS-PAGE followed by staining with Coomassie Brilliant blue. Further information about the naming system can be found in Table 1. Neg: Negative control.

In the analyses described above only active protein was monitored, but potential big differences in target gene transcript accumulation or inactive (insoluble) protein production would not be discovered by such an analysis. We therefore investigated to what extent total protein production is proportional to the amounts of transcript produced, which is not necessarily the case [43-45]. For this purpose, we included two additional proteins, the medically relevant human growth hormone (HGH) and interleukin-1RA (IL-1RA), see also (Table 2). The comparison was carried out in strains harbouring plasmids with the pMB1 replicon, which as described above generally led to a higher level of protein production (measured as activity). One general conclusion following from these experiments was that the LacI/*P*_*T7lac*_ system generated much more transcript than XylS/*Pm* (between 6.2 and 20 times more) and LacI/*P*_*trc*_ (between 3.9 and 206 times more) for all the five tested genes. XylS/*Pm* ML1-17 generated more transcript than LacI/*P*_*trc*_ as well (Figure 3), ranging from 3.3 times for *luc*_*S*_ mRNA and 88 times for *GH1*_*S*_ mRNA, except for the special case with *scFv173-2-5-phoA*. Studies of AraC/*P*_*BAD*_ was not included here since it required another host (DH10B) and since initial experiments indicated that this system (in contrast to T7) behaved very similar to XylS/*Pm* in the sense that transcript and protein amounts correlated well. At the total protein production level the analysis revealed more protein-specific effects compared to in the functional studies (Figure 2). In case of luciferase the amount of active protein was highest for XylS/*Pm* ML1-17 both according to activity measurements (see above) and deduced as soluble protein (Figure 3, Panel A). However, the very high level of transcription in the LacI/*P*_*T7lac*_ system resulted in a correspondingly big production of insoluble and inactive luciferase protein, not seen to a comparable extent for any of the other systems.

For GFP and HGH (Panels C and D) production of soluble protein was very effective in both XylS/*Pm* ML1-17 and LacI/*P*_*T7lac*_, and the final outcome at the protein level was more similar for these proteins than for luciferase. Generally, LacI/*P*_*T7lac*_ had an apparent advantage by its performance at the transcriptional level, but this potential was often not reflected at the translational level, such that the system often produced a vast amount of transcripts that were either translated into inactive protein or were not translated at all. Note also that the amounts of protein and transcript correlated well for XylS/*Pm* and XylS/*Pm* ML1-17 (except for scFv173-2-5-AP, Panel B), probably mainly because the amounts of transcript were generally much lower than for LacI/*P*_*T7lac*_ and therefore did not overload the translational machinery. It is also interesting to note that, in terms of both active and total protein produced, XylS/*Pm* ML1-17 and LacI/*P*_*T7lac*_ generally performed best. For scFv173-2-5-AP (Figure 3, Panel B) a more complex picture was observed, but this could be mainly related to the effects of toxic protein production on host growth or variability among the systems in the kinetics of induction [46].

### Uninduced expression levels are highest for LacI/*P*_*trc*_ and lowest for AraC/*P*_*BAD*_

**Figure 4:**
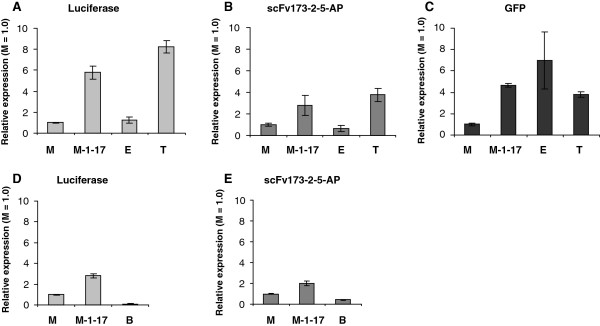
**Investigation of the tightness of different regulator/promoter systems in combination with the pMB1 replicon.** Protein activity was determined in parallel with induced cultures at the time point corresponding to five hours after induction. The data presented are from independet biological replica. The following *E. coli* strains were used as expression hosts. Panels **A**-**C**: ER2566. Panels **D**-**E**: DH10B. The capital letters represent the regulator/ promoter systems according to Table 1. Uninduced expression of GFP in DH10B was very close to the detection limit in LB medium and was left out.

The tightness of the different regulator/promoter systems is another important feature, particularly for production of host-toxic proteins [47]. We studied this with the same set-up as for induced conditions, using luciferase, scFv173-2-5-AP and GFP, and as expected the background increased for all systems when the higher copy number vectors were used. The increase was in most cases approximately proportional to that of the plasmid copy number. Therefore, only findings collected from strains harboring pMB1-based plasmids are presented (Figure 4).

Generally, LacI/*P*_*trc*_ tended to be the leakiest system producing 3.8 to 8.2 times more active protein than XylS/*Pm* under uninduced conditions. Similarly, XylS/*Pm* ML1-17 displayed 2.8- to 5.8-fold higher background expression than the wild-type system. AraC/*P*_*BAD*_ appeared to be, as expected, the tightest system giving rise to 0.1 and 0.4 times the background level for luciferase and scFv173-2-5-AP, respectively. LacI/*P*_*T7lac*_ was also quite tightly regulated although it generated the highest background expression for GFP (Figure 4, Panel C).

The ratio between the induced and the uninduced expression levels was protein dependent with relatively small induction windows for svFv173-2-5-AP (1.2-25) and large for luciferase (60–3,000). In strain ER2566, XylS/*Pm* and LacI/*P*_*T7lac*_ displayed the highest induction windows, while LacI/*P*_*trc*_ was by far the least inducible system (0.1-0.2 times compared to XylS/*Pm*). In DH10B, induction ratios for AraC/*P*_*BAD*_ were 1.3-27 times higher than the ratios of XylS/*Pm* and XylS/*Pm* ML1-17. These results are consistent with a previous report documenting that the induction ratio in the AraC/*P*_*BAD*_ system can reach up to 1,200-fold when functionally compared for the *phoA* reporter gene [11]. As for XylS/*Pm*[24,25], the induction level can also be modulated over a wide concentration range by varying the inducer concentration. In addition, uninduced levels can be even further reduced by the presence of glucose, which represses the expression in this system [47]. The main disadvantage of the AraC/*P*_*BAD*_ system is that the inducer can be metabolized in most strains of *E. coli.*

### The predicted translational efficiencies of the ribosomal binding sites vary over a wide range

**Figure 5:**
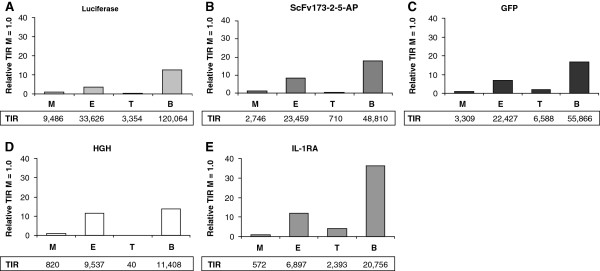
**Theoretical analysis of the translational start site by calculating the translation initiation rate (TIR).** The complete 5^′^-UTR sequences in combination with the first 50 nucleotides of the respective genes (*luc*_*S*_ (Panel **A**), *scFv173-2-5-phoA* (**B**), *gfpmut3* (**C**), GH1_*S*_ (**D**) and IL1RN_*S*_ (**E**)) were used as input sequences for the RBS calculator [54].

The DNA region corresponding to the 5^′^-UTR plays a central role in regulation of gene expression [48-50]. It covers the untranslated nucleotides at the 5^′^ end of the mRNA [51,52], including the ribosome binding site (RBS) that together with the translational start site influence expression [28,49,53]. One program frequently used to analyse the expected efficiency of these nucleotide sequences is the RBS calculator [54]. We applied its reverse engineering function on the various 5^′^-UTR-gene combinations used in the study and determined the translation initiation rate (TIR) values of the respective expression systems. The most striking finding was that the relative differences between the calculated TIRs of the four cognate RBSs were rather similar for all the five genes studied (Figure 5), although there were exceptions (see LacI/*P*_*T7lac*_ for HGH and AraC/*P*_*BAD*_ for IL-1RA). Generally, the calculator predicted that the TIR values of the LacI/*P*_*T7lac*_ and the AraC/*P*_*BAD*_ RBSs were higher than those of XylS/*Pm* and LacI/*P*_*trc*_ RBSs, suggesting a more efficient translation. The relative differences between the TIRs of the LacI/*P*_*trc*_ and XylS/*Pm* RBSs depended on the coding sequence.

To correlate the calculated TIR values with our experimental data is not straight forward because the total protein levels are obviously also dependent on the efficiencies of the promoter sequences, which are not a part of the calculation of the TIR values. However, by comparing both transcript and protein amounts available from the data presented in Figure 3 such effects can at least partly be taken into account. The amounts of accumulated transcripts derived from LacI/*P*_*T7lac*_ were generally highest and combined with a predicted more efficient TIR one might expect that this system would come out best at the protein level in all cases. However, this prediction was only in agreement with the luciferase data, and with the ScFv-173-2-5-AP and IL-1RA data to a more limited extent. In contrast, for GFP and HGH the experimental data did not support the prediction. It should also be remembered that efficient translation in itself may contribute to more accumulated transcript due to translation-mediated transcript stabilization [55,56]. For XylS/*Pm* ML1-17 there appeared to be more protein per transcript compared to LacI/*P*_*T7lac*_ and the total amounts of protein were at least equally good for this system, presumably indicating a better balance between the capacities of the transcriptional and translational systems. For LacI/*P*_*trc*_ the calculator correctly predicted a very poor expression of HGH.

In general, it is possible to some extent to use the RBS calculator to predict which regulator/promoter system would produce most protein. However, RBS function is just one among several parameters that affect the final protein production level. We have analyzed the previously reported very efficient UTR variants obtained by screening [28]. Despite the great stimulatory effect of these screened UTRs on protein expression (up to 20-fold), the calculator only predicted minor improvements relative to the wild-type sequence (between 1.5 and 3.6 times for the best variants).

### Flow cytometry analysis of GFP expression in individual cells revealed significant differences among the various regulator/promoter systems

Analyses of recombinant protein expression are mostly carried out at the level of cell populations, potentially masking significant differences in the level of expressed proteins between individual cells, which are known to occur [57,58]. If such heterogeneity exists it may represent another possibility for system improvement, e.g. by finding ways to reduce the fraction of cells with low expression level. This is also relevant in metabolic engineering projects involving metabolite flux control in biochemical pathways [59].

**Figure 6:**
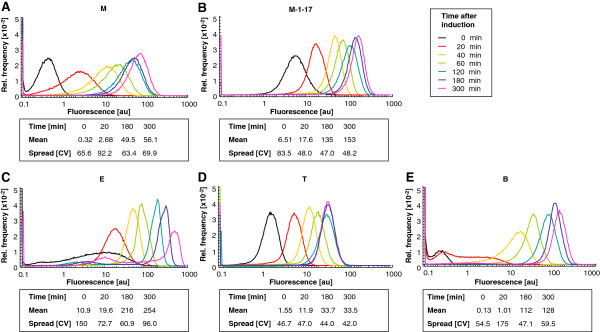
**Analysis of the distribution of expression using flow cytometry.** Strains were grown under standard conditions. At the time point of induction (t= 0 min) and at several points afterwards (t= 20–300 min), samples were collected, snap-frozen, and collectively analyzed with a flow cytometer. The spread is represented by the coefficient of variation (CV). Panels **A**-**D**: ER2566, Panel **E**: DH10B.

To analyze the level of homogeneity we used flow cytometry to quantitate GFP as it can be easily detected and because it was shown to be produced at high levels from the regulator/promoter systems used in this work, thus representing a relevant example in recombinant protein production. The fluorescence level, which reflects the number of GFP molecules, among the majority of cells harvested at a given time point typically varied in a 5–10 fold range (Figure 6). In most cases, the fluorescence values fell within a signal peak, which moved to higher intensities with extended time after induction, as expected. The highest production levels were found in cells expressing GFP from XylS/*Pm* ML1-17, *LacI/P*_*T7lac*_ and AraC/*P*_*BAD*_ (where a different host strain was used), also consistent with what was observed at the population level. However, the analysis also revealed several new observations. For the two XylS/*Pm*-based systems the distributions were broader for the wild-type system (Figure 6, Panel A) than for XylS/*Pm *ML1-17 (Figure 6, Panel B), meaning that the promoter mutations improved culture homogeneity. The reasons for this are not clear but they might be related to differences in the efficiency of transcription initiation. Fluorescence distributions of cells expressing GFP from LacI/*P*_*T7lac*_ (Figure 6, Panel C) were quite unique compared to those from the other systems. The expression profile at the time of induction is surprisingly broad in this system compared to the profiles of the remaining systems, possibly indicating low and varying (between individual cells) levels of T7 RNA polymerase production. Secondly, from two hours post induction onwards, two peaks became visible, one at rather low and one at rather high fluorescence values. The peak heights were also strongly reduced at the end. Most probably, the peak around lower fluorescence values late after induction reflects the formation of two subpopulations of cells as described by Zhao et al. [60], one being soluble GFP bearing and the other being dominated by inclusion bodies. Our findings also support those of a previous report where GFP expression was studied from a pET vector context [4].

The LacI/*P*_*trc*_ system (Figure 6, Panel D) is characterized by a very even signal distribution throughout the entire induction period. Interestingly, the mean fluorescence remained constant already two hours after induction, possibly a consequence of a very fast activation of transcription after inducer addition in this system.

The AraC/*P*_*BAD*_ system, displayed a similar behaviour as XylS/*Pm* meaning that it takes an extended time until all cells are induced as reflected by a tail of the distribution towards low fluorescence values (Figure 6, Panel E). One hour after induction, the distribution fell into a single, rather narrow peak that was shifted towards higher fluorescence values over time.

The outcomes of the flow cytometry experiments showed that there is a quite big variation in GFP expression level among individual cells. By better understanding the factors controlling this variability it may become possible to improve expression at the population level. This conclusion is supported by the observation mutations in the *Pm* promoter region lead to more homogeneity.

## Conclusions

Development of efficient recombinant gene expression protocols is often based a lot on case-specific trial and error approaches, and the results reported here contribute to the understanding of why. We have summarized the various observations (Table 3), and the LacI/*P*_*T7lac*_ system can be distinguished from all the other systems by its general tends to give rise to more transcript than all the other systems. The difference relative to the XylS/*Pm* system may be reduced or eliminated by incorporating more mutated control elements, but at the moment this would lead to very high levels of protein synthesis also in the absence of inducer [31]. Since we have shown before that even the wild-type XylS/*Pm* system can in some cases generate protein production at industrial levels it is clear that LacI/*P*_*T7lac*_ will only have an important advantage in those cases where the amount of transcript is the bottleneck. The experiments with GFP, HGH and IL-1RA illustrate cases where this has limited or no relevance (compared to XylS/*Pm* ML1-17). In contrast, for luciferase the amounts of transcript appears to be very important, but the potential in the LacI/*P*_*T7lac*_ system is in this case lost by the excessive production of inactive protein. LacI/*P*_*trc*_ generally has the advantage (for applications where this might be relevant) of a fast onset of protein production and a homogenous expression profile. However, both high levels of expression in the absence of inducer and comparatively low total production make it the least desirable if one is aiming at highest possible level of expression. AraC/*P*_*BAD*_ seems to be best with respect to tight regulation of the uninduced state coupled with high expression when induced. The XylS/*Pm* system has a big advantage of not being strain dependent in *E. coli,* and it is probably easiest to adapt to new bacterial hosts for cases where *E. coli* cannot be developed to perform in a satisfactory way. In summary we believe that the vectors developed for this study can be used as an efficient early test system for new proteins, perhaps by using XylS/*Pm* ML1-17, LacI/*P*_*T7lac*_ and AraC/*P*_*BAD*_. The outcome of such a simple first experiment will probably often lead to an identification of the nature of the main bottleneck for this particular case, shortening the time from testing to development of a good production process. The further studies would involve a detailed analysis of parameters such as growth media composition, culture incubation temperature and host strain, which are known to affect recombinant protein expression at various levels.

**Table 3:** Summary of the findings derived from the comparative expression study

**Category**	**Regulator/promoter system**				**References**
	**XylS/*Pm *and *Pm*ML1-17**	**LacI/*P***_***T7lac***_	**LacI/*P***_***trc***_	**AraC/*P***_***BAD***_	
**Components**	XylS regulator	LacI regulator	LacI regulator	AraC regulator	
	*Pm* promoter (native or variant)	*T7lac* promoter	*trp/lac* hybrid promoter	*P*_*BAD*_ promoter	
		CAP binding site		CAP binding site	
**Strain requirements**	none	strain supplying T7 polymerase	none	*araBADC*-/ *araEFGH*+ strain	
		(and lysozyme)^a^			
**Medium requirements**	none	(glucose)^b^	none	(glucose)^b^	[3,10,11,42]
**Range of inducer**	0.001 - 2.0 mM	0.05 - 2.0 mM	0.05 - 2.0 mM	0.001% - 1%	[1,25]
**Expression level**	low - high	intermediate - high	low - intermediate	intermediate - high	This study
**Basal expression**	low - high	low - high	High	low	This study
**Induction ratio**	intermediate	intermediate-high	Low	high	This study
**Accumulated transcript**	low - intermediate	high	below detection - intermediate	intermediate	This study
**RBS strength**	weak - intermediate	intermediate - strong	weak - intermediate	strong	This study
**Homogeneity**	homogeneous populations	mixed populations	homogeneous populations	mixed populations	This study
**Recommended applications**	high level expression	high level expression	(high level expression)^c^	high level expression	
	expression of toxic proteins	(expression of toxic proteins)^c^	(metabolic engineering)^c^	expression of toxic proteins	
	metabolic engineering			(metabolic engineering)^c^	This study

^a^ Expression of lysozyme, the natural inhibitor of T7 RNA polymerase, reduces the basal transcription from *P*_*T7lac*_*.*

^b^ Supplementing glucose leads to catabolite repression which reduces basal transcription levels.

^c^ Limited suitability. See ‘Results and discussion’ section for detailed information.

## Methods

### Strains, standard DNA manipulations and growth conditions

*E. coli* DH5α (Bethesda Research Laboratories) was used for plasmid propagation during cloning steps. Recombinant DH5α strains were grown at 37°C in liquid Luria Bertani (LB) broth or on solid LB plates with appropriate antibiotics (kanamycin 50 μg/mL; ampicillin 200 μg/mL). *E. coli* ER2566 (New England Biolabs, NEB) and *E. coli* DH10B (Invitrogen) served as expression hosts during the comparative studies. In comparison to the commonly used strain *E. coli* BL21(DE3), the former strain offers higher transformation efficiency for toxic clones and less background expression (NEB). All DNA manipulations were carried out according to standard procedures [61] or according to manufacturers’ instructions. PCR was performed using the Expand High Fidelity PCR systems kit (Roche), and essential regions in PCR products were verified by sequencing. Functionality of the regulator/promoter systems was confirmed using *bla* as reporter gene determining the levels of resistance to ampicillin as described previously [62].

### Vector constructions

PCR primers used during various cloning steps are listed in Table 4. Plasmids used as templates or constructs that were generated in this study are listed in Table 1.

**Table 4:** Oligonucleotides used in this study

**Name**	**Sequence (5**^′^**→ 3**^′^**)**
a) PCR primers	
Pwitw4_AscI	AAAGTGAGGGCGCGCCGGTTGATGAGAG
Pwitw5_SpeI	ATCCACCGGAACTAGTCCCCTGCTC
Pwitw6_badF	AGACTAGTAAGCCCTCCCGTATCGTAGTTA
Pwitw6_badR	TGGCGCGCCAGATGCGTAAGGAGAAAATACCG
ET_AgeI_fwd	GATGGCCCATATGATATCTCCTTCT
ET_NdeI_rev	GATCACCGGTCCAGTGATCGAA
BAD_BbsI_fwd	GGCCTTTCGTCTTCCCGGCATCCGCTTACAGACA
BAD_NdeI_rev	GACGCCCATATGTAATTCCTCCTGTTAGCCCAAAAAACG
TRC_AgeI_fwd1	TGCATGTGTCACCGGTTTTCACCGTC
TRC_NdeI_rev1	GAGCTCGAATCATATGGTCTGTTTCCTG
pelB_fwd	AGCTACATATGAAATACCTATTGCCTACG
APhis_rev2	AGGATCCGAGCCTTTCGTTTTATTGATGC
b) qRT-PCR primers	
RT-synluc_fwd2	CCATGGCTTCGGCATGTT
RT-synluc_rev2	ACACGAAAGCCGCAAATCA
gfpmut3_fwd1	CATGGCCAACACTTGTCACT
gfpmut3_rev1	CTGCTTCATGTGATCTGGGTATCT
RT-hGH.fwd1	GCCTGTGTTTTAGCGAAAGCAT
RT-hGH.rev1	AGATTGCTTTTCTGCTGGGTTT
RT-IL-1-RA.fwd1	ATTGATGTGGTGCCGATTGA
RT-IL-1-RA.rev1	TCAGACACATTTTACCACCATGAA
scFv198.fwd	GAAGGGCCGGTTCACCAT
scFv255.rev	CATTTGCAGATACAGCGTGTTCT
RT-16S-Fwd	ATTGACGTTACCCGCAGAAGAA
RT-16S-Rev	GCTTGCACCCTCCGTATTACC

Construction of pSB-M2b: The region of pBAD_gIII_calmodulin containing the origin of replication from pMB1 was PCR amplified using primer pair Pwitw6_badF and Pwitw6_badR. In parallel, pair Pwitw4_AscI and Pwitw5_SpeI was used to amplify pSB-M1b [31] excluding the RK2 *ori* (*trfA* coding region and the *oriV* origin of replication). After digestion with AscI and SpeI of both the amplified pMB1 *ori* and the pSB-M1b -resulting PCR product, the two fragments were ligated to each other resulting in plasmid pSB-M2b. The difference between copy-numbers of RK2- and pMB1-based plasmids was confirmed by agarose gel electrophoresis. Construction of pSB-P0b introducing different regulator/promoter systems: Three different regulator/promoter systems were chosen to substitute the region spanning *xylS*/*Pm* in pSB-M1b and pSB-M2b. The *lacI/P*_*T7lac*_ region was amplified from pET16b using ET_AgeI_fwd and ET_NdeI_rev and inserted into the two depicted backbones using NdeI and AgeI, generating pSB-E1b and pSB-E2b. The *lacI*^*q*^*/P*_*trc*_ region was amplified from pTrc99A using TRC_AgeI_fwd1and TRC_NdeI_rev1 prior to insertion into pSB-M1b and pSB-M2b using AgeI and NdeI, generating pSB-T1b and pSB-T2b. Finally, the PCR product covering the *araC/P*_*BAD*_ region from pBAD/gIII_calmodulin generated with the primers BAD_BbsI_fwd and BAD_NdeI_rev was inserted into the above mentioned backbones using BbsI and NdeI, creating pSB-B1b and pSB-B2b. In order to insert the *Pm* variant ML1-17 [27], pSB-M1b and pSB-M2b were digested with XbaI and PciI removing the *Pm* core promoter region which was replaced by two annealed oligonucleotides that constitute the double-stranded *Pm* ML1-17 fragment with XbaI and PciI compatible ends, creating pSB-M1b-1-17 and pSB-M2b-1-17. Introduction of other genes of interest: All pSB-P0b variants, except for pSB-B2b, were digested with NdeI and BamHI to excise the *bla* gene and to insert the *luc*_S_ gene from pIB11-luc_S_ instead, generating pSB-P0l variants. pSB-B2b and pSB-M1l were digested with NdeI and KpnI. The resulting DNA fragment corresponding to the pSB-B2 backbone and the *luc*_S_ gene were ligated to each other to generate pSB-B2l. The *scFv173-2-5-phoA* gene was PCR cloned from pHOG-173-2-5-AP with primer pair pelB_fwd and APhis_rev2. The enzyme combination NdeI and BamHI was used to replace the *bla* gene from pSB-M1b with the digested *scFv173-2-5-phoA* PCR product resulting in pSB-M1s. From there on NdeI and BamHI were used to generate all pSB-P0s variants, except for pSB-B2s. This construct was generated by digesting pSB-B2b and pSB-B1s with BamHI and ligating the pSB-B2 backbone with the *scFv173-2-5-phoA* BamHI digested insert from pSB-B1s*. gfpmut3* originating from pBAD24-GFP was inserted into the pSB-P0b variants using NdeI and BamHI with the exception of pSB-B2b. Instead, BamHI was used to excise the gene from pSB-B1g and to place it into pSB-B2 backbone (originating from pSB-B2l) to generate pSB-B2g. Genes *GH1*_S_ and *IL1RN*_S_ were excised from pMA-GH and pMA-T-IL-1RA with NdeI and BamHI and transferred to the pSB-P0b variants with the *Pm, Pm* ML1-17, *P*_*T7lac*_ and *P*_*trc*_ promoter using the same enzymes, resulting in pSB-P0h and pSB-P0r variants.

### Growth conditions for comparative expression studies

The general cultivation protocol was based on recommendations published by the European Molecular Biology Laboratory (EMBL) [63]. For *E. coli* cultivations LB medium was chosen because it is widely used among molecular biologists and at the same time it was avoided to use media with glucose as a carbon source due to the influence of glucose on background expression from *P*_*T7lac*_ and *P*_*BAD*_ through catabolite repression [64]. A growth temperature of 30°C was applied for slowing down the growth rate of *E. coli*, as this generally leads to more soluble protein [65]. Initially the kinetics of protein accumulation was studied for all expression cassettes, using GFP (fluorescence) and luciferase (activity) as the main models.The inducer concentrations and culture harvesting times post induction were varied and we found that five hours induction was sufficient to reach a plateau of accumulated protein per OD unit of cells. For GFP the accumulation rate was nearly constant (slightly lower from 3–5 hours) over this time-period. For most of the proteins it was complicated to follow the kinetics accurately since there was no quantitative method for measurement available, and in case of luciferase activity measurements may not necessarily correlate exactly with the accumulation kinetics of the insoluble fraction.

Recombinant *E. coli* ER2566 and DH10B strains were grown in 2 ml LB supplemented with 50 μg/ml kanamycin at 30°C over-night. Then 15 ml of LB with kanamycin in shake flasks were inoculated with the overnight culture to an initial OD_600_ of 0.05. Following incubation at 200 rpm and 30°C expression was induced at OD_600_= 0.5-0.6 as follows: 2 mM *m*-toluate for strains harboring *Pm*- based constructs, 1 mM IPTG for those with *P*_*T7lac*_, 0.2 mM IPTG for *P*_*trc*_ and 0.015% L-arabinose for *P*_*BAD*_. Growth was continued for 5 more hours at 30°C.

### Transcript analysis by qRT-PCR

At harvest, 0.5 ml of culture was stabilized with RNA protect (Qiagen) prior to freezing. The subsequent total RNA isolation, cDNA synthesis and relative transcript quantification by qRT-PCR was performed as described previously [28]. Primer pairs used during amplification are listed in Table 4. Transcript generated from the 16S rRNA gene was used for normalization.

### Activity measurements of the different reporters

The luciferase assay was performed using the Luciferase assay System (Promega). At harvest, the cell culture was normalized to an OD_600_ of 0.5. 90 μL of this mixture was supplemented with 10 μL of K_2_HPO_4_, pH 7.8, 20 mM EDTA prior to lysis with the Luciferase Cell Culture Lysis Reagent (CCLR, Promega). The remaining steps of the protocol were carried out according to the manufacturer’s instructions except that the luciferase activities were determined from 10 μL lysed culture mixed with 50 μL of substrate. The alkaline phosphatase assay was performed as described previously [66]. Fluorescence measurements of strains expressing GFP were performed with the FLUOstar Omega instrument (BMG Labtech) together with the corresponding Omega Software. Fluorescence intensity was determined directly from the cultures using an appropriate filter set (excitation: 485 nm; emission: 520 nm). Values were normalized against the optical density. Data were acquired from three biological and thereof three technical replica.

### Protein analysis by SDS-PAGE

For SDS-PAGE analysis 50 ml culture volume was used. Because of impaired growth of recombinant strains expressing scFv173-2-5-AP, 3xLB was used to get sufficient cell mass for analysis. The general growth conditions were as described above for the comparative expression studies. At harvest, bacterial pellets were washed with 0.9% NaCl and 100 mg pellet (wet weight) was frozen until further processing. Pellets were resuspended in lysis buffer (50 mM Tris–HCl, pH 8.0, 1 mM EDTA, 100 mM NaCl, 8 mM MgCl_2_). The solution was sonicated using a Branson Sonifier DSM tip (sonication for 3.5 minutes on ice, duty cycle 35% and output control 3.0). Soluble and insoluble fractions were separated by centrifugation and treated with 62.5 U/ml Benzonase nuclease (Merck). Protein gels were run under denaturing conditions using ClearPAGE 10% gels and ClearPAGE SDS-R Run buffer (C.B.S. Scientific) followed by staining with Coomassie Brilliant blue R-250 (Merck).

### Flow-cytometry

Cultures were grown essentially as decribed for SDS-PAGE analysis. At various time points after induction, 1 ml of culture was collected, supplemented with glycerol to 10% and snap-frozen in liquid nitrogen until further analysis. For single-cell fluorescence measurements, samples were thawed on ice and diluted in PBS. Flow cytometry was performed using the CyFlow® Space flow cytometer (Partec) equipped with a 488 nm blue solid state laser (200 mW) and a 536/ 40 nm band pass filter. For each sample, 150,000 events were collected at a rate between 800 and 2,000 events per second. Data were analysed with the Windows™ XP FloMax(R) software (Quantum Analysis). The mean and spread (coefficient of variation (CV)) of the distributions were calculated over all collected values after gating.

## Abbreviations

5′-UTR: 5^′^-untranslated region; qRT-PCR: Relative quantification real-time RT-PCR; scFv173-2-5-AP: Single-chain antibody fragment 173-2-5 alkaline phosphatase fusion protein; GFP: Green fluorescent protein; HGH: Human growth hormone; IL-1RA: Human interleukin 1 receptor antagonist; EMBL: European Molecular Biology Laboratory; SDS-PAGE: Sodium dodecyl sulfate- polyacrylamide gel electrophoresis; RBS: Ribosome binding site; TIR: Translation initiation rate; CV: Coefficient of variation; LB: Luria Bertani; NEB: New England Biolabs; IPTG: Isopropyl β-D-1-thiogalactopyranoside

## Competing interests

The authors declare no competing interests.

## Authors’ contributions

SB prepared all genetic constructs and strains, performed all experimental work on luciferase, GFP and HGH, participated in the design of the study and wrote the paper. VK performed the experimental work on scFv173-2-5-AP and IL-1RA and assisted in editing the paper. JM performed the analysis of the flow cytometry data. RL and SV conceived of the study. In addition, SV and TB participated in its design, coordinated the work and critically edited the paper. All authors read and approved the final manuscript.
